# How visual eccentricity shapes conflict via target and distractor processing in the Simon task

**DOI:** 10.3758/s13423-025-02784-5

**Published:** 2026-02-18

**Authors:** Victor Mittelstädt, Ian Grant Mackenzie, Hartmut Leuthold, Roman Liepelt, Ruben Ellinghaus

**Affiliations:** 1 Department of Psychology, University of Tübingen, Schleichstraße 4, 72076 Tübingen, Germany; 2 Department of General Psychology: Judgment, Decision Making, Action, Faculty of Psychology, FernUniversität in Hagen, Hagen, Germany

**Keywords:** Cognitive control, Action control, Conflict tasks, Simon tasks, Diffusion model, Delta plots

## Abstract

The visual Simon task is widely used to study action control in the presence of conflicting target and distractor information. However, it is unclear how one of the core parameters of this task, visual target eccentricity, affects conflict processing. Building on a quantitative dual-route model Diffusion Model for Conflict Tasks (DMC), we hypothesized that increased eccentricity alters the relative strength of target- and distractor-based activation at the stage where conflict emerges, through two mechanisms: (a) weakened target processing due to reduced perceptual quality, and/or (b) enhanced distractor processing via continuous spatial coding of location. Using DMC simulations, we demonstrate that relying solely on mean reaction time (RT) to assess eccentricity effects may obscure underlying processing differences: depending on the timing of distractor-based activation, the impact of eccentricity on mean Simon effects may vary – even when the strength of target and/or distractor processing remains unchanged. In the present two experiments, we therefore first conducted distributional (delta plot) analyses to account for the temporal dynamics of distractor processing, revealing generally larger Simon effects for far compared to near targets. Analyses of DMC best-fitting parameters showed that, in both experiments, the increased conflict with greater eccentricity was due to decreased target-based accumulation rates. Moreover, distractor-based activation increased with eccentricity in Experiment 1 but not Experiment 2, suggesting that spatial locations are coded continuously rather than categorically when they can serve as mutual reference points (e.g., within blocks). We discuss implications for the mechanisms underlying the Simon effect by elaborating on how the timing and strength of target and distractor processes jointly shape conflict dynamics.

## Introduction

One major goal of cognitive psychology is to uncover the underlying cognitive processes of goal-directed behavior amid multiple information sources. To shed light on this issue, conflict tasks are often employed. In these tasks, goal-relevant information is presented simultaneously with goal-irrelevant distracting information. For example, in the visual Simon task, participants respond to a feature of a laterally presented target (e.g., its color) (e.g., Simon, 1990). Although target location is irrelevant (i.e., distractor), participants are usually faster and more accurate when target and response location match versus mismatch (e.g., Hübner and Töbel, 2019; Liepelt and Fischer, 2016; Servant et al., 2016; Wühr, 2005). This Simon effect is commonly explained by accounts assuming that activation produced by target and distractor features are superimposed during processing, thereby facilitating (congruent trials) or impairing (incongruent trials) performance (i.e., dual-route models, cf. De Jong et al., 1994; Ulrich et al., 2015; Kornblum et al., 1990; Wühr, 2005). Furthermore, many studies explain modulations of the Simon effect (and other conflict effects) within such dual-route frameworks. For example, reduced conflict effects under heightened cognitive control have been linked to strengthened target processing (target amplification, see e.g., Scherbaum et al., 2018; Egner and Hirsch, 2005) and/or weakened distractor processing (distractor suppression, see e.g., Stürmer and Leuthold, 2003; Kelber et al., 2025; Lee et al., 2025).

**Fig. 1:**
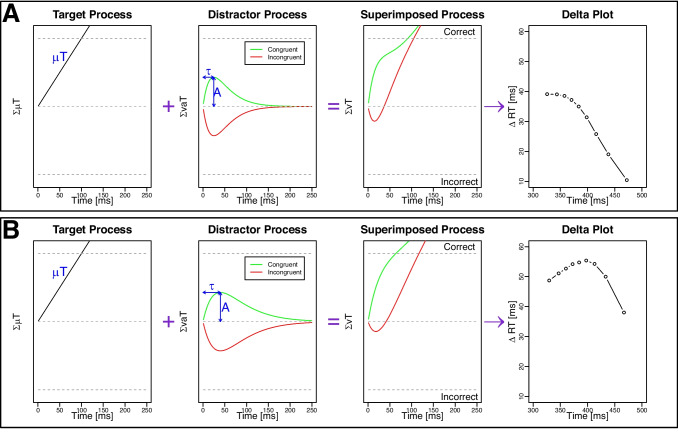
Architecture of the diffusion model for conflict tasks with delta plots illustrating varying time courses of distractor processing. Note. **A** The three panels on the left display mean activation functions based on simulations of the Diffusion Model for Conflict Tasks (Ulrich et al., 2015), implemented using the R package DMCfun (Mackenzie and Dudschig, 2021). The drift rate of the superimposed process, $\mu (t)$, results from the sum of the constant drift rate of the target process, $\mu _T$, and the time-varying drift rate of the distractor process, $\mu _D(t)$. Parameter values were as follows: $\sigma = 4$, $\mu _T = 0.7$, $\mu _R = 300$, $\sigma _R = 30$, $b = 70$, and $A = 30$. The shape parameter of the pulse-like distractor function was set to $\alpha = 2$, meaning that $\tau = 25$ ms corresponds to the time point at which the distractor-based activation amplitude *A* reached its maximum. The right panel shows the corresponding delta plot. **B** Identical to A, except that the peak of distractor-based activation *A* occurred later, at $\tau = 40$ ms

Surprisingly, however, it remains relatively unclear how one of the core parameters of the Simon task – the spatial location of the visual target (i.e., visual eccentricity) – affects conflict processing within the dual-route framework. Previous empirical findings on this issue have been inconsistent, possibly due to a predominant reliance on mean performance measures (i.e., mean reaction times and error rates), which makes it difficult to capture the multiple components involved in conflict processing (but see Yamaguchi and Proctor, 2012). To address this, we conducted two high-powered experiments (*N* = 100 each) and employed fine-grained distributional analyses (i.e., delta plots) along with diffusion model analyses to pinpoint the specific components of conflict processing that are influenced by changes in visual eccentricity.

Specifically, we based our predictions and interpretations on a quantitative dual-route model – the Diffusion Model of Conflict Tasks (DMC; Ulrich et al., 2015). The DMC extends the standard drift-diffusion model (e.g., Ratcliff et al., 2016) to the dual-route framework and describes precisely how target-based and distractor-based processes interact over time. The model assumes that the outputs of controlled (target-based) and automatic (distractor-based) processes are superimposed into a single diffusion process. As shown in Fig. 1, the drift rate at time *t* is the sum of two inputs: a temporally constant contribution from the target process, with drift rate $\mu _T$ and a time-varying contribution from the distractor process, with drift rate $\mu _D(t)$. Specifically, the DMC assumes that activation from distractor processes follows a pulse-like gamma density function, rising to a peak amplitude *A* at time $t_{\text {peak}} = (\alpha - 1) \cdot \tau$ and then falling back to zero. Given that $\alpha$ is typically fixed at 2, $\tau$ directly determines the peak time. The time for the resulting time-dependent net drift rate $\mu (t)$ to reach the decision boundary *b* reflects the decision time at the stage where the superimposed process takes place. Reaction time (RT) on a given trial is the sum of the decision time plus a normally distributed non-decision time $\mu _R$ capturing all processes outside the decision process (e.g., early stimulus encoding and late motor processes).

Critically, the key feature that *time-varying* distractor-based activation superimposes with target-based activation allows the DMC to capture the temporal dynamics of the horizontal Simon effect – something challenging for other models (e.g., Servant et al., 2014). Specifically, when moving beyond the mean Simon RT effect by plotting the difference between congruent and incongruent conditions across the RT distribution via delta plots, the Simon effect often initially increases and then decreases (e.g., Burle et al., 2013; De Jong et al., 1994; Smith and Ulrich, 2025) or monotonically decreases (e.g., Mittelstädt and Miller, 2020; Pratte et al., 2010. Several studies suggest that the time course of delta plots reflects how distractor-based activation evolves over time (e.g., Ridderinkhof, 2002; Ellinghaus et al., 2017; Tanaka et al., 2025), and modeling this with the DMC has successfully captured different delta plot patterns. As can be seen in Fig. 1, if the distractor process peaks relatively early, the Simon effect exclusively decreases with increasing response time, whereas if it peaks later the Simon effect initially increases and then decreases with time.

Because distractor-based activation varies over time, interpreting how mean Simon effects change with factors affecting processing speed – such as visual eccentricity – is generally difficult (cf. Hommel, 1993; Mittelstädt and Miller, 2020). Specifically, studies that have varied eccentricity in the Simon task usually show lower mean RT when targets are presented near compared to far from fixation (Hommel, 1993; Nicoletti and Umiltá, 1989; O’Leary and Barber, 1993; Yamaguchi and Proctor, 2019). This can be explained by reduced visual acuity at greater eccentricities impairing and prolonging perceptual processing. Thus, within the DMC, eccentricity may thus modulate the target-based drift rate $\mu _T$, as shown for other perceptual manipulations (e.g., Servant et al., 2014). Intuitively, then, one might expect that decreasing the strength of target processing with far compared to near targets should result in increased Simon effects because of a decrease in the ratio of target-to-distractor activation (cf. Mittelstädt and Miller, 2018). While some studies have indeed observed larger mean Simon effects with increased eccentricity, the findings are far from consistent. Specifically, studies have found that with increased eccentricity, the mean Simon effect sometimes becomes smaller (Hommel, 1993), larger (O’Leary and Barber, 1993; Yamaguchi and Proctor, 2019), oscillates (Lamberts et al., 1992; Roswarski and Proctor, 1996; Yamaguchi and Proctor, 2019), or hardly changes (Hommel, 1993; Nicoletti and Umiltá, 1989).

Since these studies differed in several methodological details, the discrepancies on a mean RT level may imply that somewhat different processes are respectively affected by eccentricity in these studies. However, these differences may also result from how exactly distractor processing varies over time – or at least this needs to be considered. Specifically, consider that on the one hand, increased eccentricity might (a) weaken target-based activation and hence reduce its contribution to the overall activation relative to the contribution of distractor-based activation, which should increase the Simon effect, and (b) slow down task processing, which may also decrease the Simon effect because it results in slower responses for which absolute distractor-based activation is often lower as a result of fade out (e.g., Mittelstädt and Miller, 2018). Depending on how precisely the counteracting forces of (a) and (b) combine, this could, in principle, produce all previous mean RT patterns (i.e., smaller/similar/larger mean Simon effects with increased eccentricity). However, while visual eccentricity may primarily influence the strength of target processing (i.e., target drift rate $\mu _T$), it is also plausible that other components are affected by manipulations of eccentricity.

Theoretically, one of the most relevant considerations is that the size of the Simon effect depends on the strength of target processing relative to *distractor processing* (i.e., the distractor amplitude *A*). Although most accounts assume that eccentricity is coded categorically (e.g., Cho and Proctor, 2003; Hommel, 1993; Roswarski and Proctor ; 1996), some have proposed continuous coding (e.g., Yamaguchi and Proctor, 2012), suggesting that targets at greater eccentricity could trigger stronger distractor-based activation within dual-route models like the DMC (i.e., increased distractor amplitude *A*), thereby increasing the Simon effect. For example, the Multidimensional Vector (MDV) model assumes that the stimulus – comprising both target (e.g., red color) and distractor (e.g., left spatial location) features – is represented as a single point in a multidimensional space (Yamaguchi and Proctor, 2012). The Simon effect arises from the angular misalignment between the stimulus vector – integrating target and distractor information – and response vectors encoding spatial and categorical response features: Greater misalignment leads to stronger interference. When spatial location is encoded continuously (e.g., in degrees of visual angle from fixation), more eccentric targets produce stimulus vectors with larger spatial components, increasing angular disparity and amplifying the Simon effect. While the mode of spatial coding (continuous versus categorical) may depend on factors such as the specific display configuration, the continuous coding version of the model provided the best fit to the data in a standard Simon task with varying eccentricities, which was broadly comparable to the one employed in the present study. However, unlike the DMC model, the MDV model does not separate target- and distractor-based processing over time. Instead, it combines both into a single vector before response activation, making it difficult to disentangle their individual contribution. Accordingly, in the present study, we assume that increased eccentricity may enhance distractor-based activation if target locations are coded continuously, but not if they are coded categorically within the dual-route framework.

**Fig. 2:**
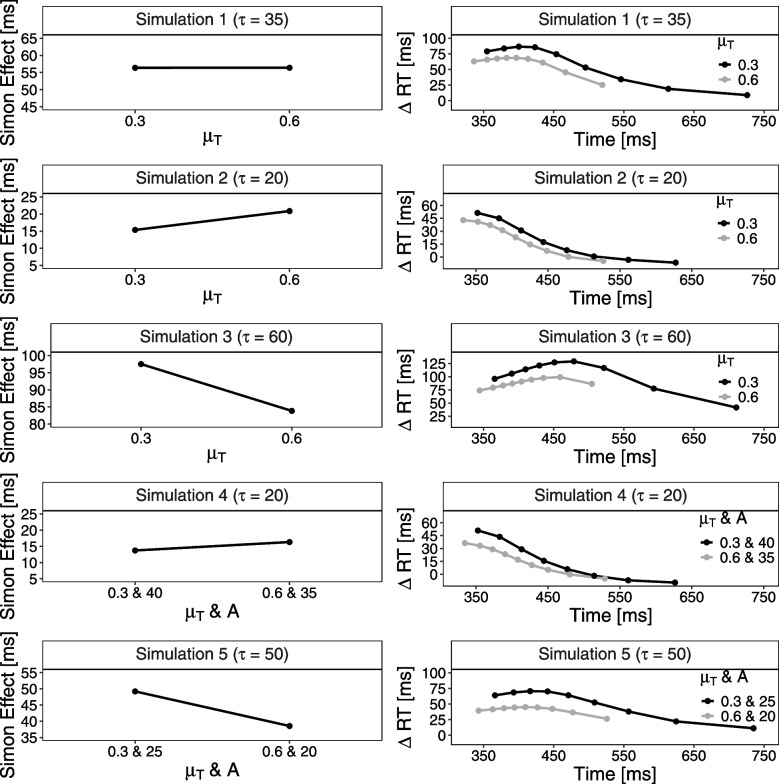
Predictions of the DMC model on a mean RT and delta plot RT level. Note. The *left panels* show mean reaction times (RTs) for congruent and incongruent trials as a function of changes in the target-based drift rate $\mu _T$ (Simulations 1–3, fixing *A* at 40), or as a function of concurrent changes in both the target-based drift rate $\mu _T$ and the distractor-based amplitude *A* (Simulations 4 and 5). The time course of distractor processing, denoted by $\tau$, varied across simulations and reflects the time point at which the distractor-related activation function reaches its maximum, because the shape parameter of the distractor function $\alpha$ was fixed at 2. For all other parameters, we used the default model parameters from DMCfun. The *right panels* show the corresponding delta plots of the simulations showing incongruent minus congruent differences in mean RTs within each of 9 percentiles

To illustrate the difficulty of uncovering the respective contributions of target- and distractor-based processing from mean RTs alone, we conducted simulations using the DMC model (see Fig. 2, for more details, see Appendix A). First, we simulated how changes in target drift rates ($\mu _T$) affect mean Simon effects under different distractor-based activation time courses, manipulating $\tau$ to vary the peak timing of distractor activation. At the level of mean RTs, all possible patterns emerged – namely, similar, reduced, or even increased Simon effects with higher drift rates – depending on the distractor time course. Only at the distributional level did a consistent pattern appear: across all RTs bin, the Simon effect was smaller with higher target drift rates. Second, to examine the potential additional contribution of distractor-based activation strength as a function of eccentricity, we ran additional simulations in which increased eccentricity was modeled by simultaneously increasing the strength of distractor-based activation (i.e., higher distractor amplitude *A*) and decreasing the strength of target-based activation (i.e., lower $\mu _T$). Again, these simulations show that different patterns of mean Simon effects can result depending on the degree of temporal overlap between target- and distractor-based activation (i.e., as modulated by $\tau$). However, consistent with the simulations that varied only $\mu _T$, the delta plot in the far-eccentricity condition again showed a downward shift when distractor amplitude *A* was also increased. Thus, these results highlight that eccentricity-related changes in superimposed processing – specifically in the strength of target- and distractor-based activation – may not be evident at the level of mean RTs, but become visible in delta plots. Still, even such fine-grained distributional analyses do not allow for a clear separation of the individual contributions of target and distractor processes, as different parameter combinations can, in principle, produce qualitatively similar delta plot patterns.

In sum, to better understand how visual eccentricity influences conflict processing in the standard visual Simon task, we conducted two experiments and went beyond the standard interpretation based on mean RT. First, we computed the delta plots for the near and far conditions. Given that the RT distributions of the two conditions should overlap, the delta plots can be compared at equal RTs, allowing for control of differences in absolute distractor-based activation that arise from the generally slower responses to far compared to near targets. If increased target eccentricity amplifies distractor-based activation and/or diminishes target-based activation, the Simon effect at equal RTs should generally be larger for far than near targets due to changes in the ratio of these superimposed activations (i.e., at each RT, the far-delta plot should be above the near-delta plot, cf. Mittelstädt and Miller, 2018). Second, we fitted DMC to the observed behavioral data, which specifies how time-varying distractor-based activation superimposes with target-based activation. These model-based analyses allow one to specifically investigate whether eccentricity influences the strength of target- and/or distractor-based activation by comparing the corresponding DMC parameters between the near and far conditions (i.e., target-based drift rate $\mu _\text {c}$ and distractor-based amplitude *A*). However, model-based analyses may also reveal effects of eccentricity on other processes – such as increased response caution (higher decision boundaries) or longer non-decision times for far versus near targets. Nevertheless, the key point remains: without a model-based approach, it is difficult to uncover how eccentricity influences processing in the Simon task within the dual-route framework.

**Fig. 3:**
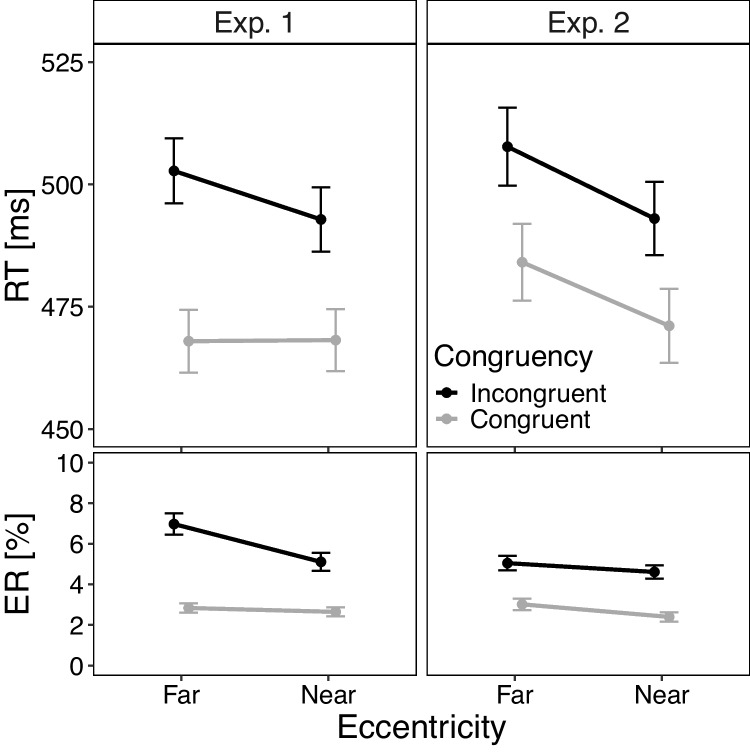
Mean reaction times (RT) and mean percentage errors (PE) in Experiment 1 and 2. Note. Mean reaction time (RT) and mean percentage error (PE) in Experiment 1 and Experiment 2 as a function of congruency (congruent, incongruent) and eccentricity (near, far). The *error bars* indicate standard errors (SE) of the corresponding means

After Experiment 1, where eccentricity varied trial-by-trial (within-block), Experiment 2 tested whether this pattern generalizes when varied blockwise (between-blocks). Such generalization is uncertain because blockwise variation may allow participants to anticipate target positions and adopt compensatory strategies – like adjusting decision boundaries *b* – to offset, for example, poorer processing of far targets, as seen in other perceptual manipulations (increasing decision boundaries in perceptually more difficult blocks, see e.g., Ellinghaus et al., 2024). Moreover, referential coding of eccentricity – based on relative spatial differences – may only occur within blocks, where eccentricities serve as mutual reference points, such that changes in distractor amplitude *A* arise within but not between blocks.

## Experiment 1

This experiment randomly varied the target eccentricity (near or far) within blocks in a standard visual Simon task.

### Method

#### Participants

One hundred participants were tested online, but the data from four participants were excluded due to accuracy below 80%. Consequently, the final sample comprised 96 people (71 women, 92 right-handed), with ages ranging from 19 to 63 years (M = 32.27).^1^

#### Apparatus and stimuli

The experiment was conducted online using the JavaScript library jsPsych (De Leeuw, 2015). All visual stimuli were presented on a grey background. A black plus sign served as a fixation point. Target stimuli were red or blue squares, which were presented on the left or right side of the screen. Note that the specific eccentricity was randomly manipulated within blocks (i.e., near vs. far). Stimulus size and distance were regulated through a calibration routine implemented at the start of the experiment. This involved adjusting the size of a rectangular shape using the mouse until it matched that of a regular bank card. Assuming participants perfectly adjusted the shape’s size during the calibration routine, the distance from the center of the target to the center of the screen was 1.6 cm in the near and 7.4 cm in the far condition. Thus, the eccentricity in the far condition was 4.6 times greater than the eccentricity in the near condition. Responses were key presses with he left and right index finger on the “Q” and “P” keys of a computer keyboard.

#### Procedure

Overall, each participant was tested in 12 experimental blocks with each block including 56 randomly ordered trials, with seven presentation of each of the eight possible stimulus displays (i.e., 2 left/right locations x 2 colors x 2 eccentricities). At the beginning of each trial, the fixation cross appeared on the screen. After 500 ms, a colored square appeared to the left or right of the fixation cross and both stimulus and fixation cross remained on the screen until participant responded, up to 2 s.After correct responses, the next trial started after the presentation of a blank screen for 500 ms. After incorrect responses, an additional error screen was presented for 1.5 s, indicating the type of error (too slow or wrong key).

#### Data preparation

The first two blocks and trials without any response were excluded from any analyses. For the behavioral RT analyses, error trials were additionally excluded. The RT delta plots were constructed by creating RT percentiles (10%, 20%, 30%,...,90%) separately for each participant within each of the four conditions (i.e., near/far $\times$ congruent/incongruent) (e.g., Mackenzie et al., 2022).

**Fig. 4:**
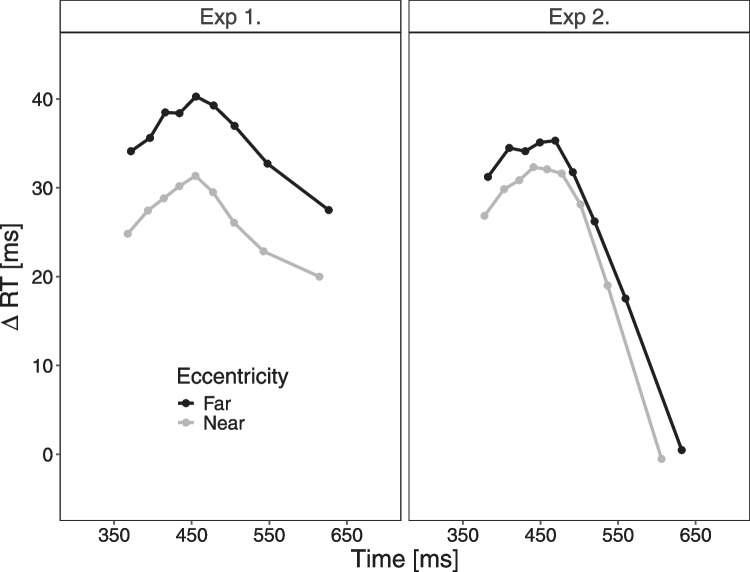
Delta RT plots in Experiment 1 and 2. Note. Delta plots in Experiment 1 and 2 showing incongruent minus congruent differences in mean RT within each of nine RT percentiles, plotted against the quantile average RTs, separately for each eccentricity condition (near, far)

**Table 1:** Best-fitting parameters of the diffusion model for conflict tasks (DMC) (Ulrich et al., 2015) to the experimental data of the two subconditions (i.e., near/far eccentricity) and the corresponding derived peak latencies of the amplitude of the distractor process as well as weighted root-mean-square errors (RMSE) averaged across participants

	Experiment 1		Experiment 2	
	Near	Far	Near	Far
*DMC best-fitting parameters*				
drift rate $\mu _T$ of target process	0.58 (0.02)	0.54 (0.02)	0.61 (0.02)	0.56 (0.02)
decision boundary $\textit{b}$	77 (2)	75 (2)	75 (2)	77 (2)
mean residual time (ms) $\mu _\text {R}$	332 (4)	332 (4)	348 (5)	349 (6)
variability residual time (ms) $\sigma _\text {R}$	29 (2)	31 (2)	30 (2)	32 (2)
shape $\alpha _\text {s}$ of starting point distribution	3.54 (0.05)	3.48 (0.06)	3.5 (0.06)	3.5 (0.05)
amplitude $\textit{A}$ of distractor process	12.9 (0.6)	16.6 (0.7)	14.5 (0.7)	14.8 (0.8)
scale (ms) $\tau$ of distractor process	103 (8)	98 (7)	85 (7)	79 (7)
Goodness-of-fit (*RMSE*)	22.2 (1.07)	23.8 (1.01)	20.9 (1.04)	23.1 (1.00)

Standard error (SE) of means in parentheses. The fitting procedure used the R-package DEoptim as implemented within the R-package DMCfun (Mackenzie and Dudschig, 2021). The step size was t = 1 ms, the diffusion constant was fixed at $\sigma$ =4, and the shape of the pulse-like distractor function was fixed at $\textit{a}$ =2

#### DMC modeling

The DMC model assumes that activation produced by target-based and distractor-based processes is combined into a single diffusion process (with a constant diffusion constant $\sigma$ = 4) that moves toward the correct decision boundary *b*. Specifically, the drift rate of this process is determined at each time point *t* by the superimposed inputs from the temporally constant drift rate $\mu _T$ of the target-based process and the time-varying input drift rate $\mu _\text {i}(t)$ of the distractor-based process. More precisely, the input from the distractor-based process is modeled as a pulse-like gamma density function with a shape parameter *a* that reaches its peak amplitude *A* at time $\textit{t}_\text {peak}= (a-1)\cdot \tau$, after which it decreases back to zero. Similar to others (e.g., Ulrich et al., 2015), the shape of this function was kept constant at *a*, so that $\tau$ directly reflects $\textit{t}_\text {peak}$. RT in a given trial is the sum of the decision time needed to reach the boundary *b* plus a normally distributed non-decision (residual) time (i.e., with $\mu _\text {R}$ and $\sigma _\text {R}$). Starting point variability is implemented by sampling from a beta-shaped distribution *B* that varies symmetrically around zero from $\textit{b}_{1}$ to $\textit{b}_{2}$.

Following previous studies (Ellinghaus et al., 2024; Mittelstädt et al., 2023, 2022), the DMC model was fitted to the observed individual data of the two experimental conditions (i.e., low/high eccentricity) from each participant using the R-package DMCfun. Specifically, the model was fitted simultaneously to the individual and condition-specific errors and RT distributions by minimizing the root-mean-squared error (RMSE) between observed and predicted values. As a fitting procedure, a differential evolution algorithm was employed (Mullen et al., 2011), and the default settings of the DMCfun fitting function were used, except that we fixed *a*=2.^2^. We then conducted paired *t* tests to compare the mean best-fitting parameters between the two conditions.

### Results and discussion

#### Behavioral performance

Figure 3 shows the mean RT and mean PE as a function of congruency and target eccentricity. A repeated-measures ANOVA with the these factors on mean RTs revealed significant main effects of congruency, *F*(1, 95) = 291.63, $p <.001$, $\eta ^2_p$ = .75, and eccentricity, *F*(1, 95) = 19.25, $p <.001$, $\eta ^2_p$ = .17. The mean RT was smaller in congruent than in incongruent trials (468 ms vs. 498 ms) and smaller with near than far targets (481 ms vs. 485 ms). Moreover, a significant interaction indicated a larger Simon effect for far (35 ms) than near targets (25 ms), *F*(1, 95) = 19.85, $p <.001$, $\eta ^2_p$ = .17.

The ANOVA on mean PEs also revealed significant main effects of congruency, *F*(1, 95) = 67.69, $p <.001$, $\eta ^2_p$ = .42, and eccentricity, *F*(1, 95) = 36.78, $p <.001$, $\eta ^2_p$ = .28. The mean PE was smaller in congruent than in incongruent trials (2.7% vs. 6.0%) and smaller with near than far targets (3.9% vs. 4.9%). The interaction was also significant, reflecting a larger Simon effect for far (4.8%) than near targets (2.5%), *F*(1, 95) = 26.29, $p <.001$, $\eta ^2_p$ = .22.

As can be seen in Fig. 4, the delta plots in both conditions followed a similar and typical time-course – that is, they were initially increasing but then decreased for larger RTs (i.e.,  460 ms). Interestingly, across the entire RT distribution, the Simon effect in the near condition was consistently smaller than the one in the far condition. This interpretation, based on visual comparison, is supported by additional delta plot analyses described in Appendix B.

#### DMC modeling

**Fig. 5:**
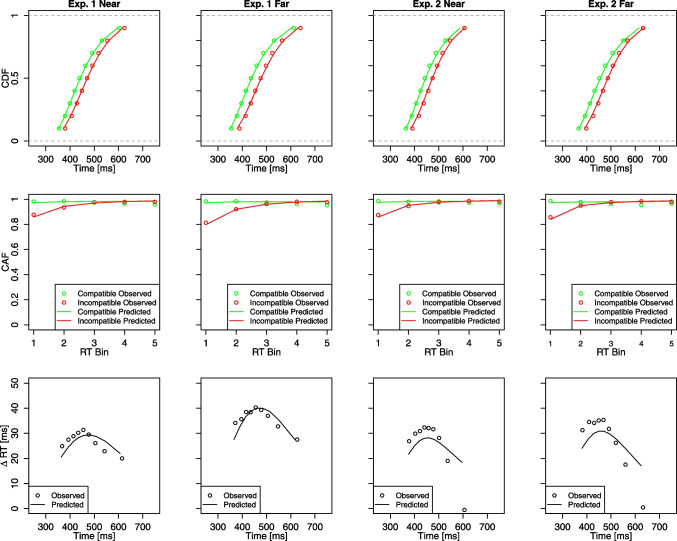
Observed values and model predictions of DMC for Experiment 1 and Experiment 2. Note. The panels within each column depict the fitting results of one eccentricity condition (i.e., near/far) separately for each experiment. The panels within each row depict cumulative distribution function (CDF) of correct RTs separately for congruent and incongruent trials, conditional accuracy functions (CAF) separately for congruent and incongruent trials, RT delta plots showing incongruent minus congruent differences in mean RTs within each of nine deciles plotted against the decile averages, respectively

The mean best-fitting parameters and mean RMSEs as a function of eccentricity are shown in Table 1. As can be seen in Fig. 5, the model fitted the distributional RT and PE data well. Target-based drift rates $\mu _T$ were significantly larger in the near compared to the far condition, *t*(95) = 4.71, *p* < .001, *d* = 0.48. Moreover, the strength of distractor-based processes *A* were significantly smaller in the near compared to far condition, *t*(95) = 5.54, *p* < .001, *d* = 0.57. No other effects were significant (all *p* > .234). Thus, the fitting results suggest that increased eccentricity leads to both stronger distractor-based and weaker target-based activation, with little, if any, influence on other processes.

## Experiment 2

Experiment 2 was designed to examine whether the pattern observed in Experiment 1 would generalize when target eccentricity varies blockwise.

### Method

Another sample of 100 participants was tested online, but the data from four participants were excluded due to accuracy below 80%. The remaining 96 people (75 women, 90 right-handed) ranged in age from 20 to 68 years (M = 35.41).

#### Participants

#### Apparatus, stimuli, and procedure

The stimuli and procedure were identical to Experiment 1, except that visual eccentricity was manipulated between blocks in an alternating fashion. Half of the participants started with low eccentricity, and the other half started with high eccentricity.

#### Data preparation

We followed the same data preparation procedure as in Experiment 1.

#### DMC modeling

Identical to Experiment 1.

### Results and discussion

#### Behavioral performance

The ANOVA on mean RTs (Fig. 3) revealed again significant main effects of congruency, *F*(1, 95) = 132.65, $p <.001$, $\eta ^2_p$ = .58, and eccentricity, *F*(1, 95) = 57.74, $p <.001$, $\eta ^2_p$ = .38. The mean RT was smaller in congruent than in incongruent trials (471 ms vs. 500 ms) and smaller with near than far targets (482 ms vs. 496 ms). Contrary to Experiment 1, the interaction was not significant ($p =.473$, $\eta ^2_p$ = .01). The Simon effect was descriptively only slightly larger for far (24 ms) than near targets (22 ms).

The ANOVA on mean PEs also revealed significant main effects of congruency, *F*(1, 95) = 57.27, $p <.001$, $\eta ^2_p$ = .38, and eccentricity, *F*(1, 95) = 7.88, $p =.006$, $\eta ^2_p$ = .08. The mean PE was smaller in congruent than in incongruent trials (2.7% vs. 4.8%) and smaller with near than far targets (3.5% vs. 4.0%). Contrary to Experiment 1, the interaction was not significant ($p =.604$, $\eta ^2_p$ < .01). Descriptively, the Simon effect was slightly smaller for far (2.0%) than near targets (2.2%).

The delta plots in both conditions followed again a similar and typical time-course – that is, they were initially increasing but then decreased for larger RTs (Fig. 4B). Note that the delta plot time-course pattern appeared similar to the one found in Experiment 1, except that the initial increase seemed somewhat less steep. Furthermore, as in Experiment 1, across the entire RT distribution, the Simon effect was smaller in the near than far condition, but this offset of delta plots was smaller in this experiment (Appendix B).

#### DMC modeling

The mean best-fitting parameters and mean RMSEs are shown in Table 1. As in Experiment 1, DMC provided a reasonable fit to the distribution of error rates and RTs (see Fig. 5), with goodness-of-fit (RMSE) values similar to Experiment 1. However, while DMC generally captured the reversed U-shape time-course, the average best-fitting parameters slightly ($\approx$ 5 ms) underestimated the Simon effects at lower percentiles. As in Experiment 1, target-based drift rates $\mu _T$ were significantly larger in the near compared to the far condition, *t*(95) = 4.03, *p* < .001, *d* = 0.41. No other effects were significant (all *p* > .277). Thus, contrary to Experiment 1, there was no evidence that the strength of distractor-based processes varied as a function of eccentricity (*p* = .602).

## General discussion

In this study, we investigated how increasing the visual eccentricity of targets in the Simon task affects conflict processing within the dual-route framework. Building on a quantitative dual-route model (DMC), we hypothesized that greater eccentricity may (a) impair perceptual processing, thereby reducing target-based activation (i.e., lowering the target-based drift rate $\mu _T$), and/or (b) enhance the salience of the distractor location due to continuous spatial coding, leading to stronger distractor-based activation (i.e., increasing the distractor-based amplitude *A*). Thus, both mechanisms would result in a higher proportion of distractor- relative to target-based activation, which should lead to increased conflict (i.e., larger Simon effects) with greater eccentricity – at least when accounting for the time-varying nature of distractor-based activation. Specifically, contrary to previous studies, we applied more fine-grained distributional (delta plots) and modeling analyses, as the Simon effect varies with response speed, making it difficult to interpret interactions in mean RTs when experimental factors like eccentricity also affect overall processing speed (i.e., faster responses to near than far targets). Specifically, DMC simulations showed that the effect of increasing eccentricity on mean Simon effects can vary–decreasing, increasing, or remaining unchanged – depending on how the timing of distractor-based activation (and thus the time course of Simon effects) interacts with changes in the strength of target- and distractor-related processing.

Regardless of whether we manipulated eccentricity on a trial-by-trial basis (Experiment 1) or blockwise (Experiment 2), delta plot analyses generally indicated a larger Simon effect for far compared to near targets when controlling for time-varying fluctuations of Simon effects (i.e., upward shift of the far relative to the near delta plot). Importantly, DMC analyses revealed that in both experiments, the stronger Simon effects with far than near targets across the RT distribution are due to decreases in the rate of target-based evidence accumulation. Furthermore, the modeling results revealed that in Experiment 1, but not Experiment 2, distractor-based activation was increased with far targets, suggesting a relative coding of target locations when different locations are intermixed. Overall, these findings provide new insights into how the spatial location of the target–a core feature of the Simon task–influences conflict processing and, more generally, highlight the interplay between the timing and strength of target- and distractor-based processes in conflict tasks.

The slowing of responses with increased eccentricity could, in principle, be attributed to processing components not directly involved in superimposed processing – such as non-decision times or decision boundaries. However, as eccentricity only systematically reduced the target-based drift rate of evidence accumulation in both experiments, this suggests that perceptual processes affected by eccentricity directly contribute to superimposed processing, where distractor-based activation interferes with the accumulation of target-related evidence. While the Simon effect has traditionally been viewed as reflecting action-related interference–occurring at the stages of response selection or initiation (i.e., locus of conflict; cf. Treccani et al., 2009; Lu and Proctor, 1994; Buetti and Kerzel, 2009)-our findings align with recent evidence highlighting the role of perceptual processes in conflict resolution within this task (e.g., Ellinghaus et al., 2024). Although superimposition could, in principle, occur at various stages (e.g., perception, response selection, or response initiation) within DMC, it is framed in terms of decision processes, as is standard for models of this type. Thus, more generally, it seems helpful to apply some caution when describing model parameters in terms of processing stages, and it may also be worth extending the DMC framework by considering that superimposition may take place at multiple stages to account for the different processing loci of Simon effects (as well as other conflict effects).

Furthermore, while most previous studies have argued that target eccentricity is coded categorically (e.g., Cho and Proctor, 2003; Hommel, 1993; Roswarski and Proctor, 1996), the present study suggests that continuous coding also needs to be considered: In Experiment 1, distractor-based activation was stronger for far than near targets when eccentricity varied within blocks, indicating more fine-grained spatial encoding. In general, this aligns with a vector-based modeling approach using continuous spatial coding, in which the stimulus is represented as a vector that integrates both target and distractor features: Greater eccentricity increases the spatial component of the stimulus vector, which can increase angular misalignment with the response vectors on incongruent trials, thereby amplifying the Simon effect (e.g., Yamaguchi and Proctor, 2012; Yamaguchi and Proctor, 2019). However, to our knowledge, this vector-based model, due to its different processing architecture, does not allow for a direct dissociation of target- and distractor-based contributions over time, which is a core feature of dual-route models such as the DMC. Interestingly, changes in distractor-based activation with eccentricity were only observed when both eccentricity levels appeared within blocks, not when varied between blocks. Potentially, the mixed design allows participants to perceive both eccentricity levels as mutual reference points. Hence, the mode of spatial coding (continuous vs. categorical) is not fixed, but shaped by global task structure – extending previous findings that coding modes can shift within blocks through display layout changes (e.g., Yamaguchi and Proctor, 2019).

The present findings also appear incompatible with temporal overlap accounts that propose eccentricity influences only early perceptual processes – before the superimposition of activation begins – thereby allowing distractor-based activation more time to develop prior to overlapping with target processing (cf. Mittelstädt and Miller, 2020; Hommel, 1993). As noted earlier, neither experiment provided evidence that eccentricity influenced non-decision time, which typically reflects processes outside the superimposition (decision) stage. Instead, our results show that target eccentricity directly impacted the strength of both target-related (Experiments 1 and 2) and distractor-related activation (Experiment 1) during the stage where these activations superimpose. However, a limitation of the current DMC is that non-decision time is modeled as a constant added to decision time, preventing selective effects on early target processes prior to superimposition. In other words, it cannot capture cases where distractor-based activation begins with target onset, but target-based accumulation is delayed due to differences in early target processes that precede the activation-superimposition stage. To address this, we conducted simulations using an modified DMC version by inserting a delay before target accumulation begins, during which the distractor-based activation already starts to decline (see Appendix C; for a conceptually related approach using a leaky accumulator model, see Wühr and Heuer, 2018). This DMC version simulates slower RTs for far targets arising solely from delays before superimposition. Although this delay-based version reduced mean Simon effects for far targets, it produced delta plots with an upward shift for near vs. far eccentricity, opposite to our observed pattern. Thus, when considering these modified DMC simulations against the actual empirical delta plot pattern (which was correctly predicted by the standard DMC simulations described in the Introduction), it further reinforces the idea that target eccentricity affects the strength of target and distractor processes, rather than *only* their temporal overlap.

Nevertheless, as the present study focused on how visual eccentricity shapes processing within the standard DMC, it remains open how other dual-route variants–with partly different mechanisms–might account for its influence (e.g., Miller and Schwarz, 2021; Luo and Proctor, 2022; Lee et al., 2024; Luo et al., 2023; López and Pomi, 2024).

## Figures and Tables

**Fig. 6:**
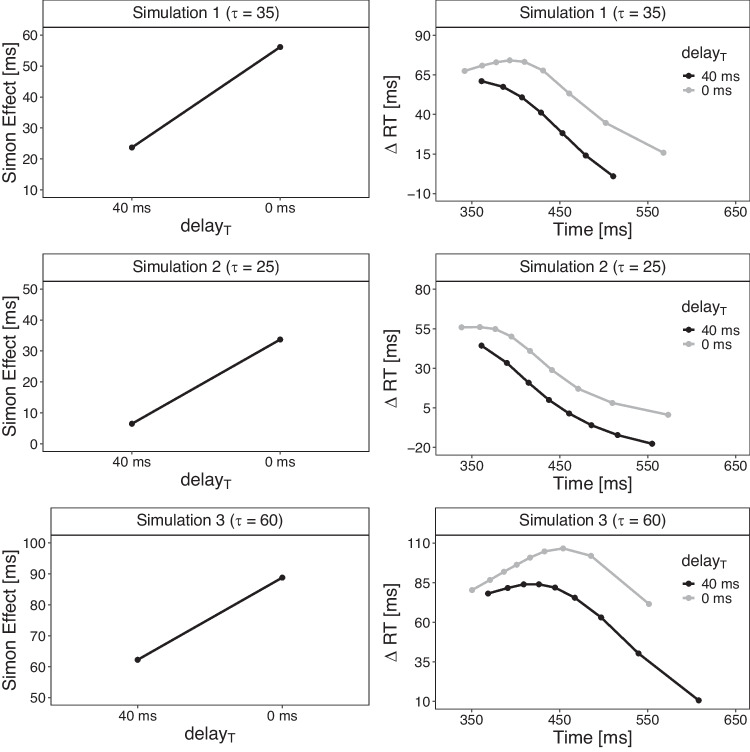
Predictions from the modified DMC with delayed target processing before activation superimposition on a mean RT and delta plot RT level. Note. The *left panels* show mean reaction times (RTs) for congruent and incongruent trials as a function of the timing of early target processes – that is, comparing conditions with $delay_T$ = 0 ms versus $delay_T$ = 40 ms – prior to the onset of target-based evidence accumulation and the subsequent superimposition of activations. The time course of distractor processing, denoted by $\tau$, varied across simulations. We used the default model parameters from DMCfun except *A* was set to 40 and we fixed *a* = 2. The right panels show the corresponding delta plots of the simulations showing incongruent minus congruent differences in mean RTs within each of nine percentiles

## Data Availability

The experiments were not preregistered. Raw data of all experiments are available via the Open Science Framework at https://osf.io/hkqxu/. Materials for the experiments reported here are available from the authors upon request.

## Appendix A Simulations with the Diffusion Model for Conflict Tasks (DMC)

First, we conducted simulations to illustrate how changes in the drift rate of target-based processes ($\mu _T$) can produce different Simon mean RT patterns, depending on the timing of distractor-based processing (i.e., $\tau$). Thus, in the Simulations 1–3, the effect of decreased target eccentricity (far vs. near) is simulated solely by increasing the drift rate of target-based processes ($\mu _T$), without changing the amplitude (*A*) of distractor-based activation.

As is illustrated in Fig. 2 (Simulation 1), the model can predict that the Simon effect on mean RT is basically independent of the relevant drift rate (with mean Simon effects of 45 ms for each drift rate). Critically, as seen in the corresponding decreasing delta plots in Fig. 2, at any given RT, the delta plots for higher $\mu _T$s are below those with lower $\mu _c$s – similar to the present experiments. Thus, the approximately equal mean RT results from two counteracting effects: Stronger target-based activation (a) decreases the Simon effect because it strengthens the contribution to the overall activation relative to the contribution of distractor-based activation, but it (b) also increases the Simon effect because absolute distractor-based activation is generally higher for faster responses (cf. Mittelstädt and Miller, 2018).

Interestingly, by using the same parameters and simply reducing the value of $\tau$ (resulting in an earlier peak of the distractor-based activation function), the mean Simon RT effect can also be higher for larger compared to smaller drift rates (i.e., 22 ms vs. 15 ms, see Simulation 2 in Fig. 2A). Notably, however, the delta plot pattern was similar – that is, the delta plots for higher $\mu _T$s were again below those with lower $\mu _T$s (see Fig. 2B). Thus, the mean RT Simon effect is again affected by these two counteracting effects described above, but in this simulation, the increases in Simon effects with faster response speed are particularly large due to the early decrease of distractor-based activation.

Finally, by increasing the value of $\tau$, the mean Simon RT effect can also be smaller for larger drift rates compared to smaller drift rates (i.e., 80 ms vs. 92 ms, see Simulation 3 in Fig. 2A). Critically, the delta plot pattern again showed that the delta plots for higher $\mu _T$s were below those with lower $\mu _T$s (see Fig. 2B). Thus, in this simulation, the decreases in the Simon effect due to strengthening the relative contribution of target-to-distractor-based activation are large enough to also decrease the mean Simon effect because the fade-out of distractor-based activation was postponed.

Second, we conducted additional simulations (Simulations 4 and 5) to examine the predictions of the DMC when modeling the effect of decreased target eccentricity by simultaneously increasing the drift rate of target-based processes ($\mu _T$) and decreasing the amplitude (*A*) of distractor-based activation. As illustrated in Fig. 2 (Simulation 4), the model can predict that the mean Simon effect on RT remains largely unchanged across these parameter combinations (with mean effects of 16 ms in both cases). However, as shown in Simulation 5, when increasing the timing parameter of distractor processing ($\tau$), the model can also predict a larger mean Simon RT effect for the parameter combination resembling far compared to near targets (i.e., 49 ms vs. 38 ms). Interestingly, Simulations 4 and 5 still yielded a pattern consistent with the previous simulations in which only $\mu _T$ was varied: Specifically, the delta plot associated with the near-target condition consistently lay below that of the far-target condition. This suggests that delta plot – but not mean RT – analyses can reliably reveal reductions in the Simon effect resulting from increases in the ratio of target- to distractor-based activation (i.e., through increases in $\mu _T$ and/or decreases in *A*), as they also account for the time course of distractor processing.

## Appendix B Additional delta plot analyses

In both Experiment 1 and 2, the delta plot in the near condition was shifted downwards relative to the delta plot in the far condition. In this appendix, we present additional statistical analyses to assess these delta plot offsets.

### Experiment 1

To compare the delta plots of the two conditions at a common value of RT (i.e., to check for the downward shift of the near condition relative to the far condition), we fitted regression lines to the delta plot for each participant and eccentricity condition separately (for a similar approach, cf. Mittelstädt and Miller, 2020): (a) for the first five bins, where the delta plots were increasing, and (b) for the last four bins, where the delta plots were increasing. We then computed the predicted Simon effect at each participant’s individual mean RT separately for the regression models computed in (a) and (b). These analyses revealed that for both (a) (28 vs. 37 ms, *p* < .001) and (b) (24 vs. 34 ms, *p* = .002), the predicted Simon effects were significantly smaller in the near condition than in the far condition. Thus, these analyses demonstrate that even when controlling for time-varying fluctuations of Simon effects, the Simon effect was significantly smaller in the near condition compared to the far condition.

We also checked for significant differences when comparing the far and near Simon effects separately at each percentile with paired *t* tests. These analyses revealed that significant differences at each percentile (all *p*s < .010) except the last (*p* = .305). Note, however, that when comparing the analyses separately for each percentile, it is not considered that there are speed differences between percentiles (i.e., the percentiles in the near condition generally have smaller RTs).

### Experiment 2

To compare the delta plots of the two conditions at a common value of RT, we fitted linear regression lines (a) for the first four bins, where the delta plots were rather increasing, and (b) for the last six bins, where the delta plots were increasing.^3^ The predicted Simon effects, calculated based on these linear fits, were again smaller in the near condition than in the far condition, with (a) (30 vs. 34 ms, *p* = .026) and (b) (21 vs. 24 ms, *p* = .245), but this reduction was only significant for (a) but not (b).

Furthermore, when comparing the far and near Simon effects separately at each percentile with paired t-tests, there were significant difference when comparing the far and near Simon effects separately at the first two percentiles (*p* = .016 and *p* = .011), marginal significant differences at the next percentile (*p* = .057), but not significant differences at the last percentiles (*p*s = .105, .115, .951, .505, .712, .890, respectively).

Thus, these analyses demonstrate that when controlling for changes in Simon effects over time, the Simon effect was, at least for faster responses, smaller again in the near condition compared to the far condition.

## Appendix C Simulations with a modified Diffusion Model for Conflict Tasks (DMC) incorporating delayed target processing before activation superimposition

In this appendix, we present additional simulations using a modified version of the DMC model, designed to capture the effect of increased target eccentricity by prolonging early target-based processing – that is, by introducing a delay ($delay_T$) before target-based evidence accumulation begins (and hence before target- and distractor-based activations are superimposed). Note that in this version of the DMC model, distractor-based processing again begins at stimulus onset, meaning that distractor activation can already evolve during the delay period ($delay_T$). For example, when $delay_T$ is very long, distractor-based activation may have already peaked and fully faded out by the time target processing begins, potentially resulting in no Simon effect – or even a reversed one – if the distractor-based activation undershoots slightly into negative values before returning to zero (Ulrich et al., 2015).

In Simulations 1–3, we modeled increased target eccentricity (i.e., near versus far) by increasing $delay_T$ from 0 ms to 40 ms. Additionally, we systematically varied the timing parameter of distractor-based processing ($\tau$) across simulations, using the same $\tau$-values as in Appendix A. As shown in Fig. 6, in all simulations, mean Simon effects were smaller with $delay_T$ = 40 ms than with $delay_T$ = 0 ms. Moreover, the corresponding delta plots were vertically shifted. However, in contrast to the simulations in Appendix A – where $\mu _T$ was varied – and also in contrast to the empirical data, the delta plots for the near-target condition ($delay_T$ = 0) were consistently shifted upward relative to those for the far-target condition ($delay_T$ = 40).

## Footnotes

1 With this final sample size, we had over 80% power to detect significant effects regarding the parameter comparisons of at least d = 0.29 at a significance level of = 0.05.

2 The results of both experiments were similar when additionally allowing *a* to vary between conditions

## Code Availability

The JavaScript code used to run the experiment and the code used to analyze the data are available upon request.
